# Standardizing XPS and HAXPES Analyses of LLZO Solid-State Electrolytes and Their Reactive Compounds

**DOI:** 10.1021/acsmaterialsau.4c00174

**Published:** 2025-06-26

**Authors:** Huanyu Zhang, Lars P. H. Jeurgens, Claudia Cancellieri, Jaka Sivavec, Maksym V. Kovalenko, Kostiantyn V. Kravchyk

**Affiliations:** † Laboratory of Inorganic Chemistry, Department of Chemistry and Applied Biosciences, 27219 ETH Zürich, CH-8093 Zürich, Switzerland; ‡ Laboratory for Thin Films and Photovoltaics, Empa - Swiss Federal Laboratories for Materials Science & Technology, CH-8600 Dübendorf, Switzerland; § Laboratory for Joining Technologies & Corrosion, Empa - Swiss Federal Laboratories for Materials Science & Technology, CH-8600 Dübendorf, Switzerland

**Keywords:** HAXPES, XPS, LLZO surface, binding energies, charge correction

## Abstract

Surface contamination of Li_7_La_3_Zr_2_O_12_ (LLZO) is a significant challenge that impedes its use as a nonflammable and nontoxic solid-state electrolyte in high energy density, temperature-tolerant Li metal solid-state batteries. This work presents detailed dual-beam lab-based XPS/HAXPES analyses of the LLZO surface, complemented by studying reference samples such as Li, Li_2_O, LiOH, Li_2_CO_3_, La_2_O_3_, ZrO_2_, and La_2_Zr_2_O_7_. The objective is to establish baseline reference data, binding energy (BE) positions and more robust chemical shifts, for unambiguously identifying potential surface contaminants and surface reaction layers, for example, as a function of the synthesis and surface treatment conditions. Furthermore, the established procedures for the calibration and charge correction of the XPS and HAXPES energy scales are proposed, as is essential for comparing results across different laboratories and for different incident X-ray sources and spectrometer setups. While lab-based HAXPES analysis of LLZO surfaces is still at its infancy, it is proven to be a very powerful tool in addition to conventional XPS for nondestructively resolving in-depth inhomogeneities in the composition of LLZO surfaces up to probing depths in the range of 20–30 nm.

## Graphical abstract

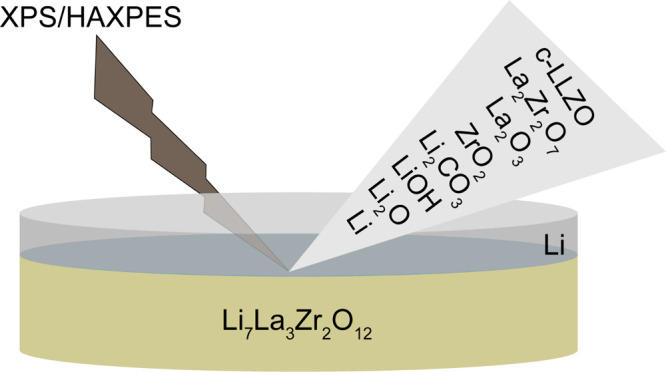

## Introduction

Li_7_La_3_Zr_2_O_12_ (LLZO) with a garnet-type structure has emerged as highly compelling Li-ion solid-state electrolyte (SSE) for next-generation Li-metal solid-state batteries (LMSSBs).^1–5^ The increased interest in LLZO stems from its superior set of properties, including high thermal and mechanical stability,^6^ high Li-ion conductivity (up to 1 mS cm^–1^ at RT),^7^ low electronic conductivity (10^–8^ S cm^–1^ at RT),^8^ and a broad electrochemical operation window ranging from 0 to 6 V vs. Li^+^/Li.^9^ However, several significant challenges must be addressed before LLZO can be effectively employed as an SSE in LMSSBs, such as (*i*) its chemical reaction with H_2_O and CO_2_,^10–17^ leading to the formation of a Li-ion resistive layer on the LLZO surface.^18,19^ (*ii*) its chemical instability with cathode materials at sintering temperatures.^20,21^ and (*iii*) challenges to maintain LLZO phase purity due to Li losses during sintering.^22^ These critical issues significantly impact the electrochemical performance of LLZO-based LMSSBs, such as increased interfacial resistance at the Li/LLZO or cathode/LLZO interfaces, resulting in high voltage polarization, as well as the formation of Li dendrites, induced by the inhomogeneous distribution of the applied current density (current focusing) at the Li/LLZO interface.

Recognizing the importance of addressing these challenges, researchers have employed various surface-sensitive techniques to investigate chemical changes occurring at the LLZO surface during successive processing steps.^23^ Among these techniques, X-ray photoelectron spectroscopy (XPS) stands out as a nondestructive, quantitative and powerful surface-selective tool for the chemical analysis of LLZO surfaces during successive stages of synthesis and postprocessing. XPS has even been performed for the *in-operando* determination of redox reactions during the plating and stripping of Li at the LLZO/Li interface.^24^ In recent years, hard X-ray photoelectron spectroscopy (HAXPES) has garnered significant attention as a complementary method to standard XPS for state-of-the-art chemical analysis of functional materials and its buried interfaces, such as those in microelectronics, batteries, catalysis and corrosion.^25,26^ HAXPES provides surface analysis with an increased probe depth of up to 10–40 nm, while being nondestructive as compared to conventional XPS sputter-depth profiling.^27,28^

Given the technological importance of resolving surface contamination issues of LLZO, as guided by surface-analytical techniques, this study presents a comprehensive combined XPS/HAXPES analyses of selected reference compounds, which are commonly encountered for research on c-LLZO, such as Li, Li_2_O, LiOH, Li_2_CO_3_, La_2_O_3_, ZrO_2_, and La_2_Zr_2_O_7_ (LZO). Importantly, in the present study, possible contamination issues due to the intermediate air-exposure of these reference compounds were minimized by performing all synthesis and postprocessing steps (e.g., annealing, sintering) in a purified Ar glovebox environment and also transferring them under an inert purified-Ar atmosphere to the ultrahigh vacuum (UHV) system for XPS/HAXPES analysis. Such an approach without intermediate air exposure is essential to provide adequate and reliable reference data on the binding energy (BE) positions and chemical shifts of the respective core-level photoelectron lines (i.e., La 3d^5^/_2_, O 1s, C 1s, Zr 3d^5^/_2_ and Li 1s) of the different chemical compounds. Notably, although many studies have reported on the XPS analysis of LLZO and its compounds, as gathered in Table 1,^29–39^ our work is the first to present the combination of XPS and HAXPES analyses of LLZO and its associated reactive compounds in a single study. As reflected in Table 1, the spread in reported BE values for a given chemical species identified for LLZO compounds typically varies in the range of 2–3 eV, which by far exceeds the estimated experimental error for the absolute BE scale of conventional XPS of about ± 0.07 eV.^40^ Such unexpected large spread in the reference BE values is certainly too some extent related to critical aspects regarding the calibration of the binding energy scale and the pros and cons of using different reference lines for charge correction, as will be carefully addressed in this paper. Additionally, we delve into other debatable issues related to the XPS/HAXPES analysis of LLZO and its compounds, such as difficulties in the unambiguous identification of chemical species due to relatively small chemical shifts with respect to the intrinsic line width, as is often encountered in the spectral reconstruction of measured O 1s and Li 1s spectra.

**Table 1:** Binding Energies (BEs) of the La 3d^5^/_2_, O 1s, C 1s, Zr 3d^5^/_2_, and Li 1s Main Peaks for LLZO and Its Compounds, As Reported in the Literature^a^

	Binding energies (eV)					
Compound	**La 3d** ^**5**^ **/**_**2**_	**O 1s**	**C 1s**	**Zr 3d** ^**5**^ **/**_**2**_	**Li 1s**	**Reference**
Li					54.24	^29^
Li					54.97	^30^
Li_2_O		531.20			56.40	^30^
LiOH		533.77			57.40	^30^
LiOH		532.8				^31^
LiOH		530.85				^32^
LiOH		532.65				^29^
LiOH		530.91				^33^
Li_2_CO_3_		534.67	292.89		58.05	^30^
Li_2_CO_3_		532.2	290.2		55.5	^34^
Li_2_CO_3_		533.7				^31^
Li_2_CO_3_		531.55	289.69		55.09	^32^
Li_2_CO_3_		533.56			55.41	^29^
Li_2_CO_3_		531.8	290.10		55.3	^35^
Li_2_CO_3_		532.05	289.84			^33^
Li_2_CO_3_		532.0	289.9			^36^
Li_2_CO_3_		532.7	289.9		56.2	^37^
Li_2_CO_3_		531.6	289.8		56.8	^38^
c-LLZO	838.6			181.3		^39^
c-LLZO		530.7		180.6		^34^
c-LLZO		530.7		182.33		^31^
c-LLZO		529.23			54.13	^32^
c-LLZO	832.38	530.7		182.38		^29^
c-LLZO	833.59	528.52			54.52	^35^
c-LLZO		528.76				^33^
c-LLZO	832	529.0		180.9		^36^
c-LLZO	834.7	529.8		182.2	55.2	^37^
c-LLZO		528.4		181.25	54.3	^38^

a The corresponding chemical species assigned to each resolved main peak are shown in the first column. These BE values should be interpreted with great care, since all values are listed as such without correcting for possible differences in the energy calibration and charge correction procedures between the different studies and laboratories. Notably, for the 3d^5^/_2_-^3^/_2_ spin-orbit doublets of La and Zr only reference BE values for the strongest ^5^/_2_-component at the lower BE side are given.

## Results and Discussion

Reference compounds of Li_2_O, LiOH, Li_2_CO_3_, La_2_O_3_, and ZrO_2_ were prepared by heat-treatment of commercially (*Cm*) purchased powders (see Experimental Section) at 100 °C overnight in vacuum oven installed in a purified-Ar glovebox, thus removing surface moisture. The bulk phase constitution of the commercially purchased powders was confirmed by in-house by powder X-ray diffraction (PXRD) (see Figure S1 in the Supplementary). The PXRD analysis indicates minor impurities of Li_2_CO_3_ and La­(OH)_3_ for the LiOH and La_2_O_3_ powders, due to reaction in air. The LZO reference powders were synthesized via solid-state synthesis, ground into powder, and subjected to the same heat-treatment process in a purified-Ar glovebox environment (see Experimental Section). To identify the impact of extended glovebox storage on the surface chemistry of highly reactive Li metal foils, two types of Li metal foil reference samples were studied: “fresh” and “stored”. The “fresh” Li sample was prepared by knife-cutting a Li metal rod (99.9% purity as purchased from Sigma-Aldrich; see Experimental Section) in the interconnected purified-Ar glovebox right before its in situ transfer to the UHV chamber for subsequent XPS/HAXPES analysis. The stored Li sample was prepared analogously and then stored overnight in the interconnected purified-Ar glovebox prior to in situ XPS/HAXPES analysis.

The LLZO sample of cubic structure (c-LLZO) was prepared by ultrafast (UF) sintering of as-pressed (green body) LLZO pellets using aluminum-doped LLZO (Al-LLZO) powder from Ampcera. This process was performed in a purified-Ar glovebox using a custom-built UF sintering setup.^41^ The PXRD of as-synthesized LZO and as-sintered c-LLZO are shown in Figure S2. We chose UF sintering over other methods for sintering LLZO ceramics, because this process can be conducted in a purified-Ar glovebox environment without intermediate air exposure steps upon subsequent XPS/HAXPES analysis (see Experimental Section for details). As such, not only intermediate air exposure steps between synthesis, processing and XPS/HAXPES analysis could be avoided, but also possible cross-contamination between the different purified-Ar glove-boxes due to e.g. organic solvent residues^23^ could be minimized. The measured XPS and HAXPES survey spectra of all reference samples, are presented in Figures S3 and S4, respectively. As reflected by the Si KLL Auger line in the HAXPES surveys, some Si contamination could be detected for the sintered powders, mainly for the Li_2_O sample, but also too much lesser extent also for LiOH, Li_2_CO_3_ and La_2_Zr_2_O_7_: see Figure S4. However, this Si contamination could be exclusively assigned to the Si-containing glue of the carbon pad used for fixing these powders due to some rest porosity. Accordingly, the XPS/HAXPES analysis areas of the powder reference samples were selected such that the characteristic Si 2*s*/2p signals from the sticky tape below the sample are not (or hardly) detected by XPS (although the more intense Si 1s and Si KLL Auger lines might still be spotted; compare Figures S3 and S4). It is thus concluded that no foreign surface or bulk contaminations are detected by XPS/HAXPES analysis of reference samples, which underlines the quality of the employed synthesis and annealing routes in a purified-Ar glovebox environment.

Before presenting the measured reference data for LLZO and its compounds, as measured with dual-beam XPS/HAXPES in the laboratory, it is important to address the adopted procedures for energy scale calibration and charge correction. First of all, all XPS/HAXPES measurements of the insulating samples were performed with a dual-beam flood gun (employing low-energy electrons and Ar ions) for charge neutralization (see Experimental Section). The most commonly adopted procedure for charge correction of XPS spectra is to reconstruct the measured C 1s spectrum by fitting synthetic components and then adjust the binding energy (BE) scale such that the lowest BE component of the C 1s spectrum (as attributed to aliphatic C–C/C–H surface species) matches a standardized value,^42^ preferably 284.8 eV^31,42^ (or 284.6 eV^32^ and 282.9 eV^43^). In a few LLZO studies, alternatively charge corrections for LLZO compounds have been adopted; e.g. the C 1s main peak assigned to C in Li_2_CO_3_ has been referenced to 289.9 eV^36^ or the Zr 3d_5/2_ main peak assigned to Zr^4+^ in LLZO has been referenced to 182.4 eV.^29^ While choosing different procedures for charge correction of measured XPS/HAXPES spectra is not inherently flawed (provided the measurement conditions and calibration procedures are accurately specified, which unfortunately is not always the case), the multitude of options poses a challenge for comparison of reported BE values and chemical shifts across publications and laboratories. Thus, there is a growing need for consensus within the scientific battery community regarding the preferred energy scale calibration and charge correction procedures for XPS/HAXPES analysis using different incident X-ray sources and spectrometer setups. In this regard, charge referencing based on the C 1s main peak from Li_2_CO_3_ is not recommended, since a Li_2_CO_3_ reaction surface layer on LLZO is typically unwanted and may not be distinguishable if the LLZO samples are stored and heat-treated under inert conditions. For example, Kravchyk et al. demonstrated a reduction in Li_2_CO_3_ surface contamination from LLZO surfaces after heat treatment at temperatures ranging from 600 to 900 °C under inert conditions, as revealed by XPS analysis.^35^ Also, the suitability of the Zr 3d_5/2_ peak is questionable due to the prevalent contamination of LLZO surfaces with Li_2_CO_3_ and/or LiOH, rendering the Zr 3d_5/2_ peak from the bulk LLZO lattice often undetectable due to the limited probing depth in the range of 5–7 nm for Zr 3d photoelectrons excited by Al–Kα X-rays from LLZO and its compounds: see Table S1. We therefore propose to apply the common charge correction procedure of the fitted aliphatic C–C/C–H peak, located at the lower BE side of the C 1s peak envelope to the recommended reference value of 284.8 ± 0.2 eV,^42^ while ensuring an accurate assessment of the chemical shifts of all remaining adventitious carbon species in the reconstructed C 1s spectral envelop. Adventitious carbon (Adv-C) surface species arise from the spontaneous adsorption (physisorption and chemisorption) and/or reaction of carbon-containing gas-phase species with the surface and typically result in the coexistence of different classes of aliphatic Adv-C surface species of e.g. the alkyl-type (C–C/C–H), the organic-oxygen-type (e.g., C–O=C, C–O, and O=C) and/or the carbonate-type (CO_3_).^42^ The formation of such Adv-C species are unavoidable upon ambient exposure and can only be suppressed to some extent when working in a purified Ar-glovebox environment (while working with organic solvents^23^). Only a true UHV working environment (with a base pressure <10^–8^ Pa) may prevent the formation of such Adv-C species. Still an improved reproducibility of the sample synthesis, storage and handling procedures can be achieved when working in an Ar-purified glovebox environment (by suppressing such surface contaminations with respect to air exposure), as performed in the present study. Admittedly, other more dedicated methods have been proposed to correct for (differential) charging issues of insulating samples, such as decorating the sample surface with a noble metal, like Au, while referencing to the well-defined Au 4f^7^/_2_ peak position.^44,45^ However, such approaches are cumbersome, may induce chemical modifications to the original surface (e.g., Au may react with Li metal, forming Li–Au alloys) and may introduce additional surface contamination and/or impurities. We therefore propose a more practical approach that can be widely adopted by the battery research community, i.e. charge correction of the resolved aliphatic Adv-C 1s peak to the recommended reference value of 284.8 ± 0.2 eV.^42^ As concluded in a recent in-depth study,^42^ the commonly applied Adv-C charge referencing methodology for 1237 diverse insulating samples gave satisfactory and meaningful results in 95% of the 522 cases assessed. Nevertheless, the recommended C 1s charge correction procedure should merely be regarded as a practical tool for aligning different measurement series, since absolute BE values might be invalidated by differential charging effects and also critically depend on e.g. the applied energy calibration procedures of the XPS instrument (see above). To suppress differential charging issues during XPS/HAXPES measurements of heterogeneous nonconductive samples (possibly leading to local work function differences), the use of a (dual-beam) floodgun is a mandatory first requirement to arrive at a solid chemical-state analysis of the recorded data set. In this regard, it is emphasized that such differential charging issues may especially be significant for e.g. insulating powders and oxidized metals (differential charging can be generally neglected for bulk insulating samples, provided the sample is lifted from ground). As verified in the present study, XPS and HAXPES analysis of the oxidized/reacted Li metal foils with and without lifting the samples from ground resulted in the same binding energy values, peak shapes and chemical shifts; this indicates that any differential charging effects should be negligibly small. As a necessary next step, an accurate assessment and confirmation of the resolved chemical shifts between the different local chemical states of a given element in the studied compounds and/or reaction layers should be performed. In this regard, it is emphasized that the different probing depths of XPS and HAXPES provide an extremely powerful tool to circumvent any (destructive) sputter-cleaning steps and instead nondestructively reveal different chemical species within and below surface contamination/reaction layers (see what follows).

In the following, some general notes on the energy scale calibration procedures for XPS and HAXPES analysis using different incident X-ray sources and spectrometer setups should be made. The International standard ISO 15472,^40^ as employed for the energy scale calibration of conventional XPS instruments using monochromatic Al–Kα radiation (*h*ν = 1486.7 eV), gives an accuracy of ± 0.07 eV for the determination of absolute BE values. Up to date, no ISO standard for the energy calibration of HAXPES analysis using e.g. Cr–Kα radiation (*h*ν = 5414.7 eV; i.e. with an extended energy scale of up to 5400 eV) has been reported. However, a calibration procedure for the extended energy scale of HAXPES, which heavily leans on the ISO standard ISO 15472 for conventional XPS, has recently been proposed in ref 27. Application of the proposed energy calibration procedure for HAXPES in ref 27 results in an estimated error of ±0.17 eV for the absolute BEs measured with Cr–Kα radiation (with a corresponding accuracy in the linearity of the HAXPES energy scale smaller than <0.01%): see Experimental Section and Figures S5 and S6. Accordingly, all BE values measured and presented in the present study have an estimated accuracy in the range of ± 0.1 eV (for XPS) to ± 0.2 eV (for HAXPES using Cr–Kα radiation).

The C 1s, O 1s, Li 1s, La 3d^5^/_2_ and Zr 3d^3^/_2_:3d^5^/_2_ spectra, as measured with XPS and HAXPES, from all reference compounds were charge corrected as mentioned above and subsequently fitted with symmetric Gaussian–Lorentzian synthetic peak shapes to resolve the chemical species for each photoelectron line, while applying the same fitting constraints to the entire data set (for details, see Experimental Section). The resulting reconstructions of the charge-corrected C 1s and O 1s XPS and HAXPES spectra for *commercial samples* (*Cm*), i.e. fresh Li, stored Li, Li_2_O, LiOH, and Li_2_CO_3_, are shown in Figures 1a and 1b, respectively. The corresponding C 1s and O 1s spectral reconstructions for the *in situ prepared* Li references, i.e. Ar-sputtered Li, *in situ* formed Li_2_O, and *in situ* formed LiOH, are shown in Figures 2a and b, respectively. The corresponding C 1s and O 1s spectral reconstructions for La_2_O_3_, ZrO_2_, LZO and c-LLZO are shown in Figure 3a and b, respectively. The spectral reconstructions for the Li 1s XPS and HAXPES spectra of *all* commercial and in situ prepared Li reference samples are shown in Figure 4. Finally, the spectral reconstructions of the La 3d^5^/_2_ and Zr 3d^3^/_2_:3d^5^/_2_ XPS and HAXPES spectra of La_2_O_3_, ZrO_2_, La_2_Zr_2_O_7_ and c-LLZO are shown in Figure 5a and b, respectively. The corresponding BE values of the fitted main peaks in the reconstructed La 3d^5^/_2_, O 1s, C 1s, Zr 3d^3^/_2_:3d^5^/_2_, and Li 1s XPS and HAXPES spectra, as pertaining to the different chemical species resolved for each reference compound, are tabulated in Table 2.

**Figure 1:**
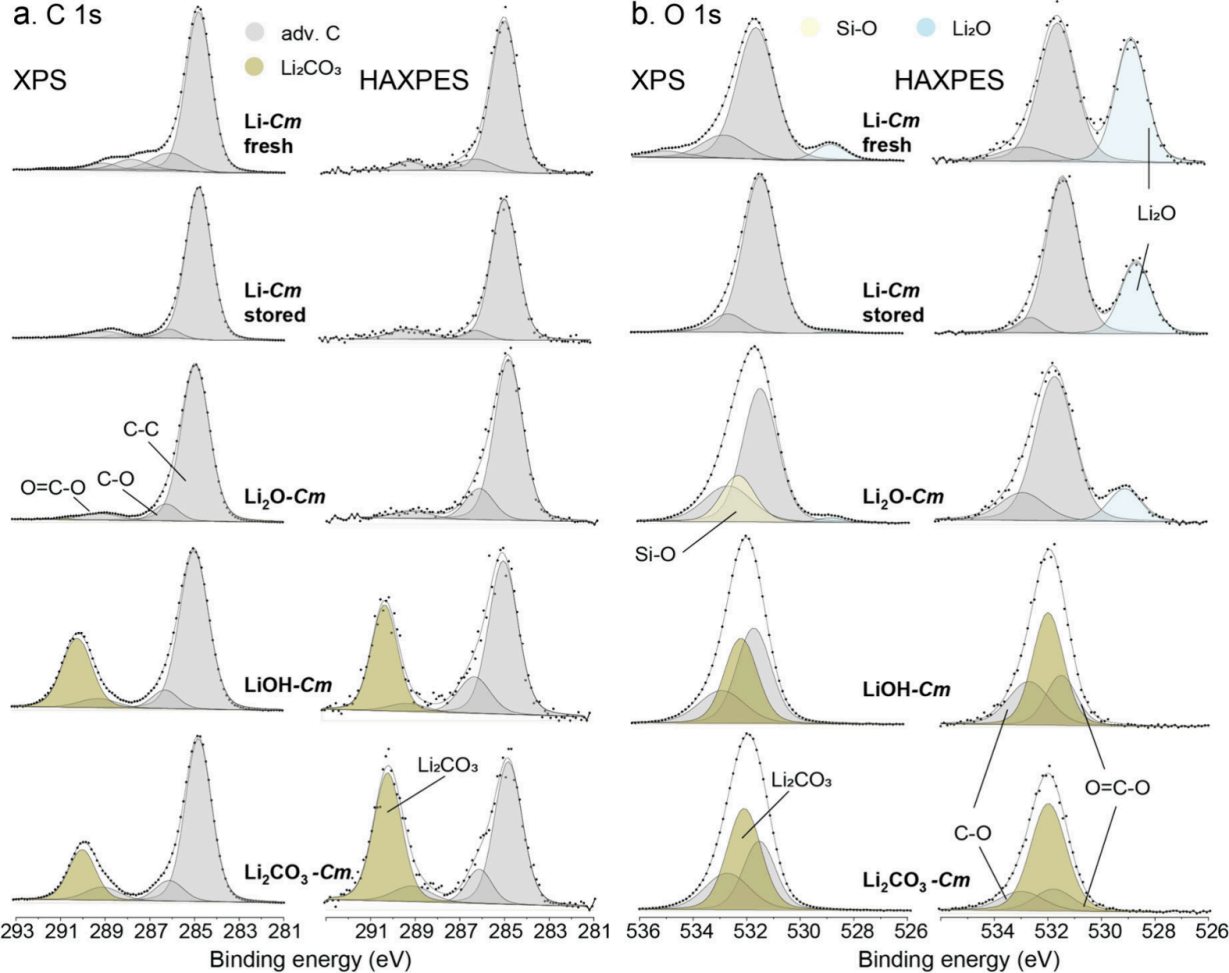
As-measured and reconstructed (a) C 1s and (b) O 1s spectra of the commercial (*Cm*) reference samples, including fresh Li, stored Li, Li_2_O, LiOH, and Li_2_CO_3_, as obtained by XPS and HAXPES. Note that the different Adv-C species in the reconstructed C 1s and O 1s are all indicated in gray.

**Figure 2:**
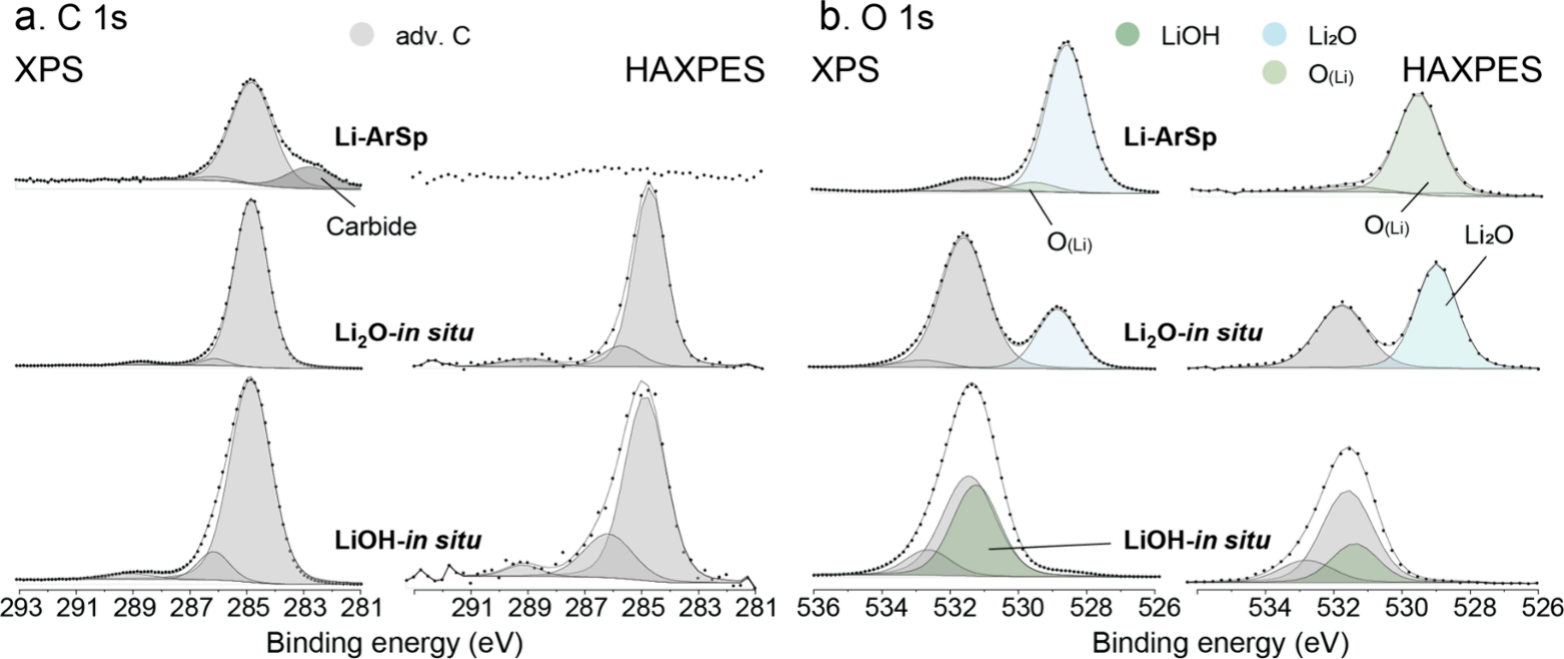
As-measured and reconstructed (a) C 1s and (b) O 1s spectra of the in situ prepared reference samples, Ar-sputtered (ArSp) Li, *in situ* formed Li_2_O, and *in situ* formed LiOH, as obtained by XPS and HAXPES.

**Figure 3:**
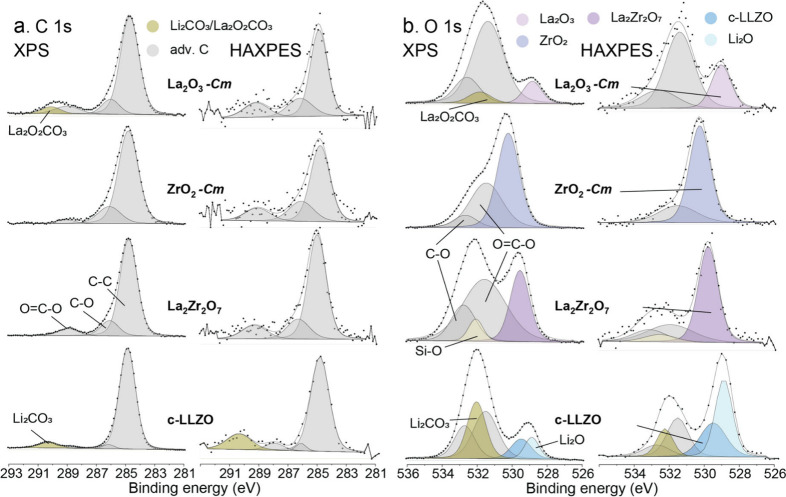
Measured and reconstructed (a) C 1s and (b) O 1s spectra of La_2_O_3_, ZrO_2_, LZO and c-LLZO, as obtained by XPS and HAXPES.

**Figure 4:**
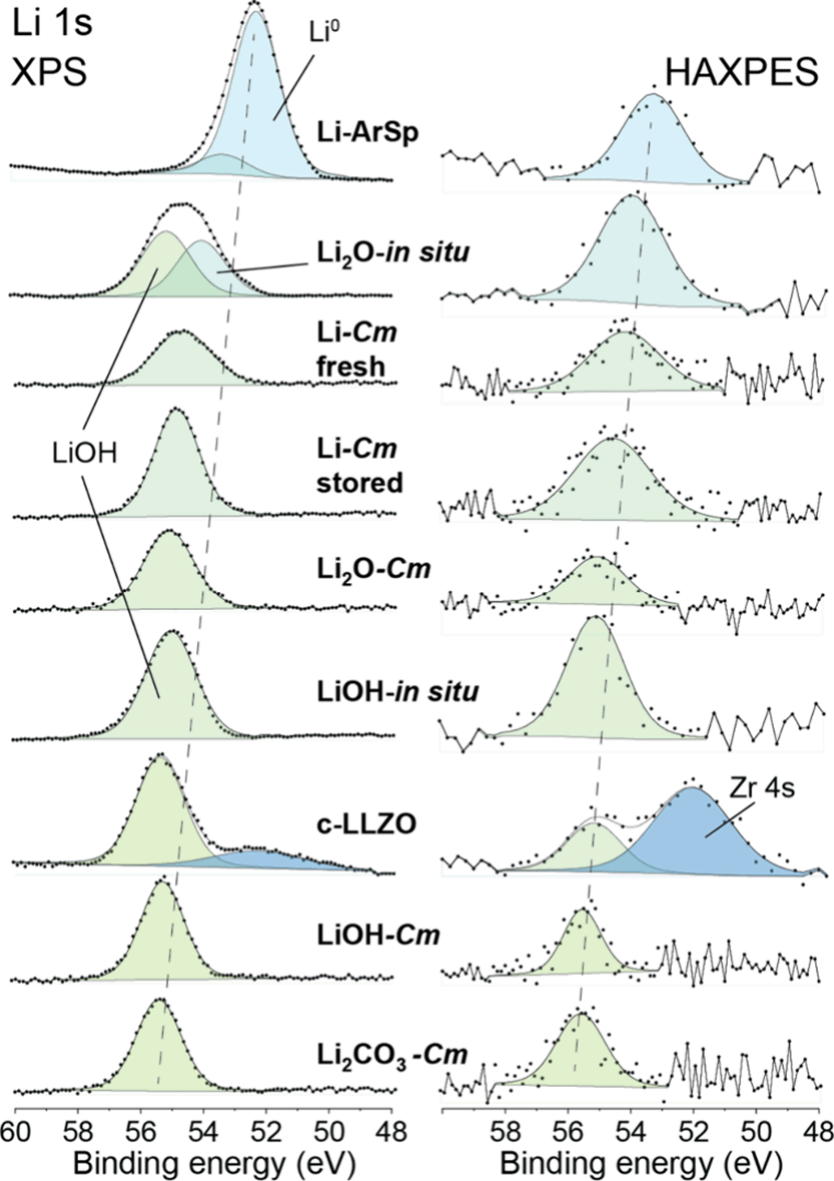
Measured and reconstructed Li 1s spectra of fresh Li, stored Li, Ar-sputtered Li, Li_2_O, *in situ* formed Li_2_O, LiOH, *in situ* formed LiOH, Li_2_CO_3_ and c-LLZO, as obtained by XPS and HAXPES.

**Figure 5:**
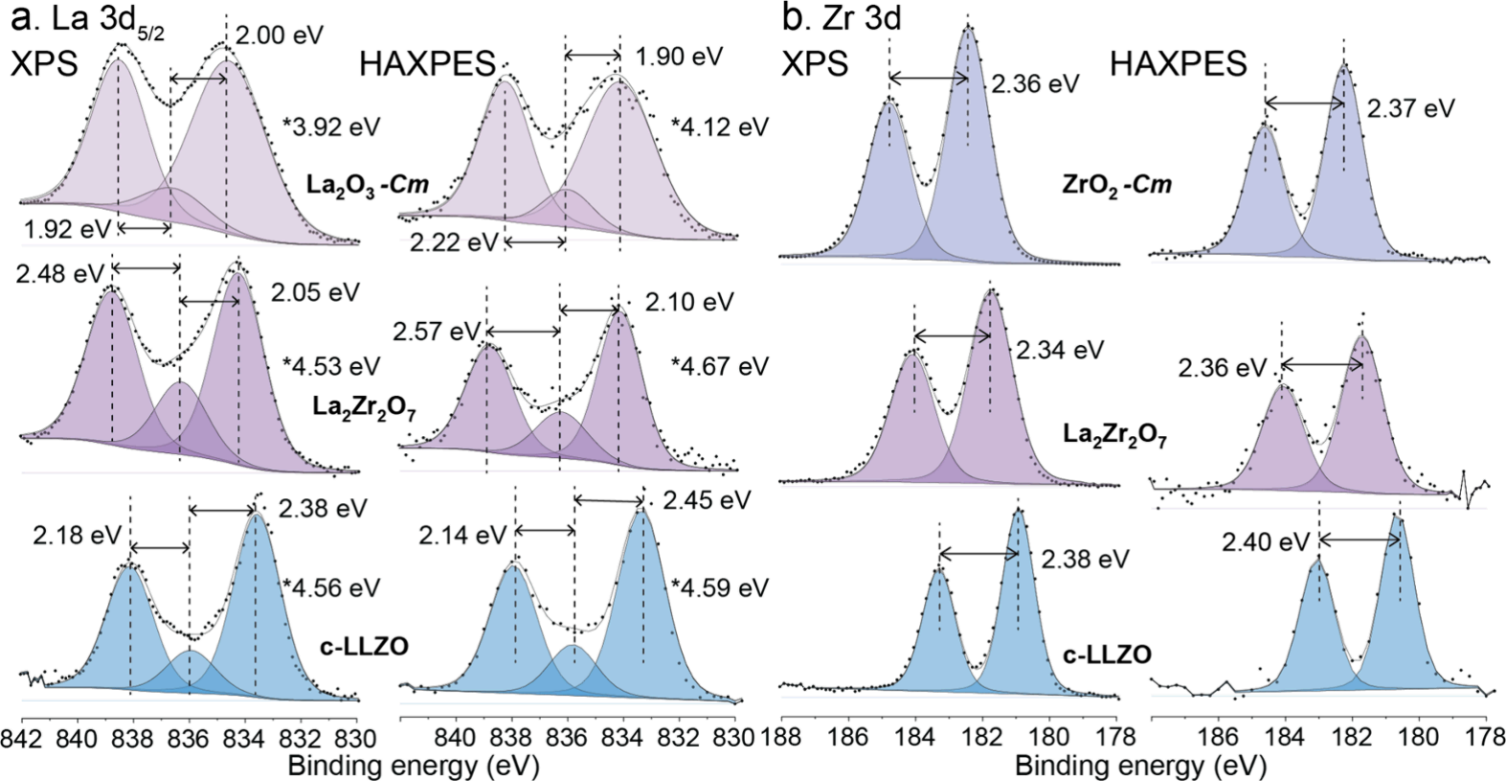
Measured and reconstructed (a) La 3d^5^/_2_ and (b) Zr 3d^3^/_2_-3d^5^/_2_ spectra of La_2_O_3_, ZrO_2_, LZO and c-LLZO, as obtained by XPS and HAXPES. *Indicating the BE difference between f^0^ and f^1^L_b_ components of each La 3d^5^/_2_ spectra.

**Table 2:** Binding Energies (BEs) of the Fitted Main Peaks in the Reconstructed La 3d^5^/_2_, O 1s, C 1s, Zr 3d^3^/_2_:3d^5^/_2_, and Li 1s XPS and HAXPES Spectra Pertaining to Figures 1–5, as Resolved by Constrained Peak Fitting of the Charge-Corrected Spectra of All Reference Samples^a^

	Binding energies (eV)								
		La 3d^5^/_2_					Zr 3d		
Chemical State	X-ray Source	f^0^	f^1^L_ab_	f^1^L_b_	O 1s	C 1s	3d^5^/_2_	3d^3^/_2_	Li 1s
Li-*Cm*	Al–Kα								54.78
	Cr–Kα								54.19
Li Ar sputtered	Al–Kα				530.37				52.26
	Cr–Kα				530.25				53.28
Li_2_O-*Cm*	Al–Kα				528.84				55.12
	Cr–Kα				528.94				55.07
Li_2_O *in situ*	Al–Kα				528.79				53.97
	Cr–Kα				528.77				53.97
LiOH-*Cm*	Al–Kα				532.04				55.31
	Cr–Kα				531.98				55.55
LiOH *in situ*	Al–Kα				531.29				55.02
	Cr–Kα				531.29				55.22
Li_2_CO_3_-*Cm*	Al–Kα				532.09	290.08			55.42
	Cr–Kα				532.01	290.23			55.59
La_2_O_3_-*Cm*	Al–Kα	834.59	836.59	838.51	528.84				
	Cr–Kα	834.12	836.02	838.24	529.03				
ZrO_2_-*Cm*	Al–Kα				530.22		182.42	184.78	
	Cr–Kα				530.26		182.25	184.62	
La_2_Zr_2_O_7_	Al–Kα	834.24	836.29	838.77	529.58		181.75	184.09	
	Cr–Kα	834.13	836.23	838.79	529.78		181.71	184.07	
c-LLZO	Al–Kα	833.57	835.95	838.13	529.49		180.93	183.31	55.37
	Cr–Kα	833.36	835.81	837.95	529.48		180.66	183.06	55.17
C–C (adv. C)	Al–Kα					284.80			
	Cr–Kα					284.80			
C–O (adv. C)	Al–Kα				531.50	286.10			
	Cr–Kα				531.50	286.10			
O–C=O (adv. C)	Al–Kα				532.70	289.10			
	Cr–Kα				532.70	289.10			

a The corresponding chemical species assigned to each resolved main peak are shown in the first column. The La 3d^5^/_2_ main peak corresponding to the final state without charge transfer is denoted as f^0^, whereas the two associated satellite peaks for the bonding and antibonding component of the final state with charge transfer are denoted as f^1^L_b_ and f^1^L_ab_, respectively. All BE values reported here have an estimated accuracy in the range of ± 0.1 eV for XPS (using monochromatic Al–Kα radiation) and ± 0.2 eV for HAXPES (using monochromatic Cr–Kα radiation): see Experimental Section and Figures S3 and S4. The corresponding FWHMs of the fitted components can be found in Table S4 of the supplementary material.

Comparable spectral contributions of the different chemical species in the reconstructed XPS and HAXPES spectra for a given reference sample hint at a relatively homogeneous in-depth composition. Or in other words, any compositional in-depth inhomogeneity should result in differences in the relative areal intensities of the fitted peak components for the reconstructed XPS (surface sensitive) and HAXPES (more bulk sensitive) spectra. Accordingly, it can be concluded from comparison of the C 1s and O 1s XPS and HAXPES spectra in Figures 1–3, that, except for the Li_2_O, LiOH, Li_2_CO_3_, La_2_O_3_ and ZrO_2_ reference samples, all other reference samples exhibit significant variations in chemical composition within the probing depth range of a few (XPS) to tenths (HAXPES) of nanometers (see Tables S1 and S2). The in-depth compositional heterogeneities of these reference samples are most evident from comparison of the reconstructed XPS and HAXPES O 1s spectra. For example, a pronounced O 1s peak component at the lower BE side of the O 1s peak envelop for the Li foils is only detected by HAXPES: see Figure 1b. The different chemical species identified by surface-sensitive XPS and more-bulk-sensitive HAXPES analysis will be discussed in the following.

The C 1s main peaks at BE values of 284.8, 286.1, and 289.1 eV, which are resolved for all references samples studied, can be attributed to the coexistence of C–C, C–O, and O–C=O Adv-C surface species, respectively (cf. gray colored components in reconstructed C 1s and O 1s spectra in Figure 1). This corresponds to chemical shifts of 1.3 ± 0.2 eV and 4.3 ± 0.2 eV of the C 1s main peaks of C–O and O–C=O with respect to the aliphatic C–C main peak (as charged-referenced at 284.8 ± 0.2 eV; see above). The two O-containing Adv-C main peaks assigned to C–O and O–C=O give rise to two corresponding O 1s chemical species at 531.5 and 532.7 eV, respectively. The fresh Li sample shows an additional (tiny) Adv-C component, as tentatively assigned to C=O surface species, giving rise to a C 1s component at 287.83 eV and a corresponding O 1s component at 534.83 eV. Here it is emphasized that the chemical shifts of the fitted Adv-C peaks with respect to the C–C peak at 284.8 eV serve as important criteria to validate the Adv-C charge correction procedure. The resolved O 1s main peak at 532.4 eV, which is most evident for LZO in Figure 2, could be assigned to Si–O bonding, as originating from sticky silicon-containing carbon pads (see above discussion pertaining to the HAXPES survey spectra in Figure S4). This stresses the importance to check for potential signal intensities from the underlying substrate materials when analyzing powders with rest porosity, especially for HAXPES.

The commercial Li foil samples, Li_2_O, and *in situ* formed Li_2_O display a pronounced O 1s chemical component at around 528.7 to 528.9 eV (see Figure 2b), which clearly originates from Li–O chemical bonding of Li_2_O. This O 1s main peak is not only particularly pronounced for the HAXPES analysis of “fresh” and “stored” Li metal, but also distinct for the Li-ArSp and Li_2_O-in situ samples (corresponding to a probing depth as deep as 39 nm; see Table S2). This implies that, even short exposure of Li metal in Ar-filled glovebox (with >0.1 ppm of oxygen gas partial pressure), leads to some oxidation of the highly reactive Li metal surface. To improve the reference quality for “clean” Li metal, the freshly prepared Li samples were in situ Ar-sputter-cleaned in the XPS/HAXPES chamber (sputter depth of roughly 30 nm), revealing an additional O 1s component at a lower BE of 530.3 eV. The peak is most pronounced for the HAXPES analysis (probing depth of 39 nm), which suggest O contamination in the bulk Li metal foil. Hence, the Li/Li-oxide interface cannot be assumed to be atomically abrupt but is instead more gradual (i.e., with a gradient of the O concentration from the surface across the Li/Li-oxide interface into the bulk metal). This rationalizes the appearance of substoichiometric Li_2_O_*x*_ (*x* < 1) oxide phases and/or dissolved O in the Li metal (as probed at larger depths by HAXPES), which complies with its slightly higher binding energy (corresponding to a lower ionicity) as compared to O^2–^ in Li_2_O. The appearance of substoichiometric oxidic and dissolved O species at metal/oxide interfaces are very common,^46,47^ being more pronounced with increasing solubility of O in the metal. The O 1s component associated with dissolved O atoms in the bulk Li metal gives rise to an O 1s main peak at 530.3 eV and is designated as O_(Li)_ in Figure 2. Bulk impurities of O in pure Li metal are only detectable by HAXPES and cannot be ruled-out in commercially supplied Li metal due to the very high reactivity of solid, liquid and evaporated Li with O, even under UHV conditions (as is the case for other highly reactive metals like Ti, Al and Mg).^48^ Consequently, if commercial Li foils are used, the possible presence of a tail of dissolved O within the lithium metal foils itself should be verified, which is more easily detectable using Auger depth profiling.^49^

Several other conclusions can be drawn from careful examination of the O 1s and C 1s spectra of “fresh” and “stored” Li metal in Figure 1. Both the XPS and HAXPES spectra of “fresh” and “stored” Li metal exhibit significant signals due to adventitious carbon species (i.e., O–C=O, O–C and C–C), despite the freshly cut Li surface being exposed to a purified-Ar glovebox environment (<0.1 ppm of H_2_O and <0.1 ppm of O_2_) for only a few minutes. Adv-C surface contamination is a common issue in battery research due to the extensive use of carbon-based additives and organic electrolytes during synthesis, even when working in a purified-Ar glovebox environment.^23^ The purified-Ar glovebox, which is directly connected to the UHV chamber for XPS/HAXPES analysis, was solely used for mounting powder/pellet samples for XPS/HAXPES analysis and never for chemical synthesis (using e.g. organic solvents) and/or postprocessing (e.g., sintering). This observation strengthens our argument for using the lower-BE C 1s peak at 284.8 eV for charge referencing of LLZO and its reactive compounds, since it seems practically unavoidable.

Next, we have to point out an interesting aspect regarding the chemical assignment of the O 1s main peak at 532.0 eV, as resolved for LiOH, Li_2_CO_3_ and c-LLZO: see Figures 1b and 2b. In the literature, it is widely accepted that this peak cannot be unambiguously assigned to Li_2_CO_3_ (provided that an associated C 1s peak is observed at 290.2 eV) but rather originates from a phase coexistence of Li_2_CO_3_ and LiOH. Our combined XPS-HAXPES analysis from the LiOH and Li_2_CO_3_ references in Figures 1 and 3 also cannot distinguish between them in the measured O 1s, C 1s and Li 1s XPS and HAXPES spectra. Moreover, the reconstructed C 1s and O 1s spectra for Li_2_CO_3_ and LiOH, as recorded at different probing depths (by XPS/HAXPES), are also extremely similar. This suggests that the commercially supplied LiOH powders (with the bulk phase constitution confirmed by XRD; see Figure S1a) have an initial surface shell of Li_2_CO_3_ with a thickness that exceeds the HAXPES probing depth in the range of 30–35 nm (see Table S2). Therefore, the “reference” XPS and HAXPES spectra of the sintered LiOH reference powders, as presented in Figure 1 and 3, should be interpreted with great care. Although XRD analysis confirm a LiOH bulk phase constitution (see Figure S1a), the surface of the commercial LiOH powders have reacted to Li_2_CO_3_ (despite careful handling in this study). In other words, a bulk phase analysis like XRD cannot be used to validate the powder quality of such reactive commercially purchased compounds. To obtain a high-quality reference for LiOH, an *in situ* formed LiOH reference sample was prepared by reaction of fresh Li metal with deionized (DI) water in the Ar-purified glovebox: see Figure 2. As a result, an O 1s peak component could be resolved at 531.3 eV and unambiguously assigned to the O in LiOH (i.e., at a slightly lower BE position as compared to O in Li_2_CO_3_ at 532.0 ± 0.2 eV; see Table 2).

Distinguishing between Li_2_CO_3_ and LiOH by analyzing the corresponding Li 1s spectra in Figure 3 is equally challenging, since the chemical shift of the Li 1s line for different Li compounds is also very small. Still, tiny differences in both the BE and the full-width-at-half-maximum (fwhm) of the Li 1s main peak can be resolved between the different Li compounds, which can be attributed to variations in the bond ionicity. For example, Li metal should correspond to the lowest BE, while Li in Li_2_CO_3_ should have the highest BE, in accord with the observed Li 1s chemical shifts in Figure 4. Notably, a Li 1s chemical shift between *in situ* formed LiOH and Li_2_CO_3_ could be resolved, but not between LiOH-*Cm* and Li_2_CO_3_ (due to the thick Li_2_CO_3_ overlayer on the LiOH-*Cm* sample). As reflected in Figure 4, the fwhm of the Li 1s peaks for fresh and old Li foil are larger than the corresponding fwhm’s of the more ionic Li-compounds, which becomes especially evident for the increased probing depth achieved by HAXPES. The relatively broad Li 1s peak widths for fresh and old Li foil can be primarily attributed to the in-depth inhomogeneity of the pure Li foils (probing both metallic and different oxidic states of Li, especially for HAXPES; see above). A Li 1s BE position for metallic Li could only be obtained by extensive sputtering of the Li foils under UHV conditions (resolving a metallic Li 1s peak at 52.3 eV), which certainly induced sputter damage; moreover, some bulk oxygen impurities in the Li metal still remain (see above). Hence, the expected *asymmetric* Li 1s peak shape, as reported for pure Li metal in ref 50, could not be revealed in this study. We therefore refrained from peak fitting the corresponding Li 1s spectra with two arbitrary synthetic peak components (i.e., with an asymmetric metallic and a symmetric oxidic synthetic peak shape of different fwhm). Still, the expected chemical shift of the Li 1s peak from lower to higher BEs for Li foil to Li_2_O/LiOH/Li_2_CO_3_ is clearly observed and can be used for a qualitative chemical state analysis (without arbitrary fitting): see Figure 4.

Notably, the Zr 4s peak of LLZO partially overlaps with the Li 1s peak, which might easily be overlooked, depending on the thickness of the LLZO surface contamination layer. The larger probing depth of HAXPES (compare Tables S1 and S2) results in a more pronounced Zr 4s spectral contribution: see Figure 4. In this regard, it is important to note that any metallization layers applied to the LLZO surface to mitigate Li dendrite formation may result in a spectral overlap of the Li 1s peak with relatively broad metallic plasmon peaks from the valence band region, as encountered for e.g. thin Sb metallizations on LLZO.^28^ So depending on the LLZO stack configuration under study, an overlap of different spectral contributions in the Li 1s region might complicate its deconvolution into the individual spectral components. It is therefore recommended, as a first step, to analyze the relationships between the peak positions and fwhm’s of the recorded Li 1s spectra and the bond ionicity, instead of arbitrarily deconvoluting the spectral envelope into individual spectral components with tiny chemical shifts. Acquiring the spectra at different probing depths by combining XPS and HAXPES and/or using different angles of detection might be very helpful in unravelling different spectral contributions in the relatively crowded lower BE range up to a binding energy of 50 eV.

As emphasized in the literature,^50–52^ especially for light atoms like Li and C, a shift and slight asymmetric broadening of their respective Li 1s and C 1s peaks toward higher BEs with increasing photon energy due to the so-called recoil effect should be expected. The recoil effect arises from the fact that the emitted photoelectron “kicks” the core-ionized atom from which it is ejected in accordance with the conservation of momentum, giving rise to a loss of its kinetic energy (resulting in an apparent increase of the BE of the respective photoelectron line).^50–52^ Accordingly, the peak position of the C 1s and Li 1s main peaks of any given chemical species, as measured by HAXPES (*h*ν = 5414.7 eV) should be shifted to higher BE values with respect to the same main peaks resolved by XPS (*h*ν = 1486.7 eV). Recent studies on the recoil effect for the Li 1s photoelectron lines indicate a recoil shift of less than 0.1 eV for a variation of the incident photon energy in the range from 1.5 to 5.5 keV,^53^ which is smaller than the estimated error in the HAXPES BE scale of ±0.17 eV in the present study (see above). Consequently, a possible shift of the Li 1s peak components due to the recoil effect cannot be resolved in the present lab-based dual-beam XPS/HAXPES study. However, for the much better energy resolution achieved at modern synchrotron facilities, such recoil effects might need to be considered. Since carbon is heavier than Li, any recoil shift of the C 1s peak excited using Cr Kα X-ray radiation in the laboratory is neglected here.

Next, the reconstructed O 1s spectra for c-LLZO in Figure 3a will be shortly discussed. The reconstructed O 1s spectra of c-LLZO, as measured by XPS and HAXPES, indicate contributions from O in the c-LLZO lattice and O in Li_2_O for the lower-BE side of the O 1s envelop: see also Figure S7 in the Supporting Information. Strikingly, HAXPES probes a relatively higher content of O from Li_2_O with respect to O from the c-LLZO lattice, as investigated in greater detail with depth-profiling XPS as a function of the sintering treatment in refs 54 and 55 . Clearly, combining a lab-based soft and hard X-ray source in the XPS/HAXPES analysis of c-LLZO and its compounds increases the confidence in resolving and distinguishing the different chemical species as a function of their depth below the surface. Since the probing depth of the HAXPES analysis using Cr Kα X-ray radiation typically exceeds the depth of the sputter-induced mixing zone (when applying low Ar^+^ sputter voltages of, say, ≤1 keV), the unperturbed chemistry at deeply buried interfaces can still be resolved by applying HAXPES sputter-depth profiling.^28^ The O 1s spectral contribution from O in the c-LLZO lattice can also be easily distinguished from that of O in ZrO_2_ and O in La_2_Zr_2_O_7_, especially when comparing the reconstructed O 1s spectra in Figure 3b with the respective Zr 3d^3^/_2_:3d^5^/_2_ spectra in Figure 5b. As reflected in Figure 3b, the O 1s main peaks of ZrO_2_ (530.3 eV) and LZO (529.8 eV) are positioned at higher BEs with respect to the O 1s main peak of O in the c-LLZO lattice (529.5 eV). On the contrary, the O 1s main peak of La_2_O_3_ (529.0 eV) is positioned at a lower BE with respect to that of O in the c-LLZO lattice (529.5 eV). The BE shifts of Zr 3d peak of ZrO_2_ and LZO compared to c-LLZO are very similar to the observed trend for the corresponding O 1s main peaks (compare Figures 3b and 5b) with the Li-containing metal oxide c-LLZO having the lowest BE (due to stronger electronegativity of Zr with respect to Li). Hence, O in the c-LLZO lattice is well distinguishable from O in ZrO_2_ and La_2_Zr_2_O_7_ by careful analysis of the O 1s and Zr 3d photoelectron lines. The chemical shifts of the O 1s main peak for the different O 1s chemical species, as resolved in this study, with respect to the resolved BE position for O in Li_2_CO_3_ at 532.0 eV, are tabulated in Table 3. The relative BE shifts of O 1s peaks can serve for internal calibration of the energy scale in case an aliphatic Adv-C signal cannot be detected (such as in sputter-depth profiling^55^).

**Table 3:** Chemical Shift of the O 1s Main Peak for the Different Chemical Species of O with Respect to the Resolved BE Position for O in Li_2_CO_3_ at 532.0 eV

Compounds/bonds	BE – BE(Li_2_CO_3_) (eV)
**Li**_**2**_ **O** *in situ*	–3.24
**Li**_**2**_ **O**-***Cm***	–3.16
**La**_**2**_ **O**_**3**_-***Cm***	–3.12
**c-LLZO**-***Syn***	–2.57
**La**_**2**_ **Zr**_**2**_ **O**_**7**_-***Syn***	–2.37
**O**_**(Li)**_ **in Li metal**	–1.95
**ZrO**_**2**_-***Cm***	–1.81
**LiOH** *in situ*	–1.04
C–O (adv. C)	–0.55
**LiOH**-***Cm***	–0.14
**Li**_**2**_ **CO**_**3**_-***Cm***	0
O–C=O (adv. C)	0.65

In the following, the resolved peak components for the isolated La 3d^5^/_2_ spectral component of the 3d^3^/_2_:3d^5^/_2_ spin–orbit doublet (see Figure 5a), as well as for the full 3d^3^/_2_:3d^5^/_2_ spin–orbit doublet, are discussed (see Figure 5b). The Zr 3d region for ZrO_2_, La_2_Zr_2_O_7_ and c-LLZO can be well described by two main peaks representing the Zr 3d^5^/_2_-3d^3^/_2_ spin–orbit doublet, which indicates a single chemical state of Zr in these samples (i.e., corresponding to Zr cations in the bulk lattice of the respective compound, as Zr is not contained in any of the reactive overlayers). The splitting of the Zr 3d^3^/_2_:3d^5^/_2_ doublet (as indicated in Figure 5b) for ZrO_2_, La_2_Zr_2_O_7_ and c-LLZO is very similar (about 2.4 ± 0.1 eV, in accord with ref 56) and is therefore not sensitive for phase identification in the present study. Spectral reconstruction of the measured La 3d^5^/_2_ peak for La_2_O_3_, La_2_Zr_2_O_7_ and c-LLZO was achieved by introducing three synthetic main peaks, as physically identified and resolved in ref 57, i.e. the La 3d^5^/_2_ main peak at the lower BE side of the spectral envelop corresponding to the final state without charge transfer (denoted as f^0^), as well as two satellite peaks for the bonding and antibonding component of the final state with charge transfer (denoted as f^1^L_b_ and f^1^L_ab_): see Figure 5 and Table 2. As discussed in e.g. ref 57, the energy splittings between the ground-state and the bonding and antibonding La 3d^5^/_2_ components can serve as sensitive fingerprints for phase identification of different La-containing compounds. This is confirmed in the present study by a difference in the corresponding splittings for La_2_O_3_, La_2_Zr_2_O_7_ and c-LLZO, as denoted in the reconstructed La 3d^5^/_2_ spectra of the reference compounds in Figure 5a. No constraints were applied to the fwhm or BE positions of the f^0^, f^1^L_b_ and f^1^L_ab_ components for peak fitting of the La 3d^5^/_2_ envelop, as imposing such constraints could obscure sensitive fingerprint information. However, the well-separated BE positions of the fitted the f^0^ and f^1^L_ab_ components should not depend much on the fitting constraints and thus provide a robust fingerprint for the chemical state of La. Interestingly, La_2_O_3_ shows slightly different splittings for XPS and HAXPES, indicating the possible reaction of the La_2_O_3_ surface with moisture in air. Indeed, a small spectral contribution of La_2_O_2_CO_3_ due to the reaction of La_2_O_3_ with CO_2_ is evidenced in the reconstructed C 1s and O 1s spectra of La_2_O_3_: see Figure 3 (with a C 1s main peak at 290.1 eV and an O 1s main peak at 531.9 eV). Else, the XPS and HAXPES Zr 3d^3^/_2_:3d^5^/_2_ and La 3d^5^/_2_ spectra for La_2_O_3_, ZrO_2_, La_2_Zr_2_O_7_ and c-LLZO have very similar shapes (reconstructions) and can all be fitted with a single chemical species, representative for the respective cation in the bulk lattice: see Figure 5a and 5b, respectively. This indicates a relatively homogeneous depth-distribution of the chemical states of Zr and/or La cations in the studied reference compounds.

Finally, as inspired by ref 30, we also report the resolved energy differences between the “reference” main peak component, as resolved from the reconstructed La 3d^5^/_2_, C 1s, Zr 3d^3^/_2_:3d^5^/_2_, and Li 1s spectra, and the corresponding “reference” O 1s main peak component resolved from the corresponding reconstructed O 1s spectra: see Table S5. These relative energy difference should be independent of the absolute BE scale calibration (and only depend on the calibration of the linearity of the BE scale). The energy differences in Table S5 may thus serve as a robust method for cross-checking the consistency of the chemical state analysis across the recorded core–shell regions (independent of the applied charge correction procedure).

## Conclusions

Although XPS analysis of LLZO surfaces has been performed in numerous studies on Li-garnet solid-state batteries, the interpretations of the XPS spectra of LLZO ceramics remain highly controversial, which is mainly due to its very high reactivity with the ambient. As convincingly demonstrated in this study, a bulk phase analysis by e.g. XRD cannot serve as a robust measure to validate the quality of commercially purchased reference samples due to the extremely high surface reactivity of most Li-containing compounds. We reiterate that commonly reported XPS analysis of LLZO-based batteries lack standardization in the charge correction procedure, which hampers quantitative comparisons and impedes progress in resolving surface contamination issues of LLZO. State-of-the-art HAXPES analysis of LLZO surface is still at its infancy but is shown to be a very powerful in addition to conventional XPS for nondestructively resolving in-depth inhomogeneities in the composition of LLZO surfaces up to probing depths in the range of 20–30 nm.

In this context, we have reported a comprehensive XPS and HAXPES analyses of the c-LLZO surface, along with reference samples such as Li, Li_2_O, LiOH, Li_2_CO_3_, La_2_O_3_, ZrO_2_, and LZO, all measured without intermediate air exposure between thermal treatment and analysis in a purified-Ar atmosphere, as well as by in situ preparation methods for Li, Li_2_O and LiOH. This provides baseline reference BE data for the different chemical species encountered, as summarized in Tables 2 and 3, which includes surface impurities and reaction layers. We propose and experimentally demonstrate the benefits of applying a charge correction of the BE scale such that the lowest BE component of the reconstructed C 1 s spectrum (as attributed to aliphatic C–C carbon) matches a standardized value of 284.8 ± 0.1 eV. Calibration of the energy scale should be based on ISO standard ISO 15472, while the energy calibration procedure for HAXPES can by adopted from ref 27.

Acquiring the core-level photoelectron spectra at different probing depths by combining XPS and HAXPES (and/or using different angles of detection) is shown to be very helpful in unravelling the different spectral contributions, especially in the relatively crowded lower BE range up to a binding energy of 50 eV, containing the Li 1s photoelectron line. If commercial Li foils are used, the possible presence of substoichiometric Li-oxide species and/or dissolved O within the bulk of the lithium metal foils may occur. Commercially supplied LiOH powders may have a thick initial surface shell of Li_2_CO_3_ with a thickness that exceeds the HAXPES probing depth. In fact, only our in-house in situ prepared LiOH and Li_2_O reference samples could reveal tiny Li 1s and O 1s chemical shifts between LiOH, Li_2_O and Li_2_CO_3_. Oxygen in the c-LLZO lattice is well distinguishable from O in ZrO_2_, La_2_Zr_2_O_7_ and c-LLZO by careful analysis of the O 1s and Zr 3d^5^/_2_ photoelectron lines. Lastly, we highlight that although the presence of La_2_O_3_, La_2_Zr_2_O_7_ and c-LLZO is typically deduced from the analysis of the O 1s spectra of LLZO, these phases can be more easily and unambiguously identified from the splittings between the La 3d^5^/_2_ ground state peak and its bonding and antibonding satellites.

## Experimental Section

### Chemicals

Li rod (Sigma-Aldrich, rod, 99.9%), Li_2_O (Alfa Aesar, 99.5%), LiOH (Sigma-Aldrich, anhydrous, 99.9%), Li_2_CO_3_ (Sigma-Aldrich, battery grade, 99.9%), La_2_O_3_ (Sigma-Aldrich, 99.99%), ZrO_2_ (Sigma-Aldrich, 5 μm, 99%).

### Ar Sputter Cleaning of Li Metal

Li fresh sample was sputtered with a focused 2 keV Ar beam in the XPS/HAXPES chamber, rastering an area of 2 × 2 mm^2^ for 3 min, corresponding to a sputtering depth of roughly 30 nm.

### Preparation of *in Situ* Formed Li_2_O

*In situ* formed Li_2_O sample was prepared by oxidizing fresh Li in 20 mTorr of oxygen flow in a vacuum chamber with base pressure below 10^–8^ mTorr for 10 min. The annealing chamber is vacuum-connected to XPS instrument without exposing to air, as described in our previous study.^58^

### Preparation of *in Situ* Formed LiOH

A Li fresh sample was reacted with deionized (DI) water in Ar-filled glovebox directly before transferring to the XPS chamber. In order to remove dissolved CO_2_, O_2_ and N_2_, the DI water was heated to 100 °C for 30 min and cooled down to room temperature under constant Ar purging. The DI water was transferred into the Ar-purified glovebox and dropped on the surface of Li fresh samples with subsequent transfer into a HV load-lock for introduction into the UHV analysis chamber.

### Synthesis of La_2_Zr_2_O_7_

To synthesize La_2_Zr_2_O_7_, the stoichiometric amount of La_2_O_3_ and ZrO_2_ were mixed in acetone (1 mL for 1 g of solid) and ball milled for 3 h at 300 rpm (with a 1:15 ratio of solid to ball mass). The resulting mixture was then dried overnight under vacuum at 100 °C and pressed into 15 mm pellets using a uniaxial pressure of 5 tons. The pellets were then heated in a tube furnace at 1500 °C for 12 h under O_2_ flow and ground to powder for further XPS/HAXPES measurements.

### Preparation of Sintered c-LLZO Sample

240 mg of aluminum-doped LLZO powder with a nominal composition of Li_6.25_Al_0.25_La_3_Zr_2_O_12_ (Al-LLZO powder from Ampcera) was loaded into a pressing die and uniaxially compressed with a force of *ca*. 10 kN. Subsequently, the surface of the green body pellets was carefully polished using SiC abrasive paper to remove any visible impurities. To remove moisture and Li_2_CO_3_ from the LLZO surface, the pellets were then dried on an alumina plate at 200 °C for 30 min in air, followed by a heat-treatment at 900 °C for 10 min in an Ar-filled glovebox using a sacrificial LLZO pellet as a substrate. Then, the pellets were ultrafast-sintered at 1200 °C for 120 s with preheating step at 1000 °C for 20 s (see ref 41 for details). The phase constitution of the received powder and the sintered LLZO pellet were analyzed by powder XRD, as presented in Figures S1 and S2, respectively.

### Material Characterization

**Combined XPS/HAXPES analysis** was performed using a PHI Quantes spectrometer (ULVAC-PHI) equipped with a monochromatized soft Al–Kα (*h*ν = 1486.6 eV) and a monochromatized hard Cr–Kα (*h*ν = 5414.7 eV) X-ray source. The XPS/HAXPES analysis chamber is directly coupled to a glovebox with an oxygen and water purifier system, as used for accepting air-sensitive samples under a shielded Ar atmosphere through a load-lock system. The sensors in the purified-Ar glovebox indicate a water and oxygen content of <0.2 ppm of H_2_O and <0.2 ppm of O_2_, respectively. To prevent the glovebox from being contaminated with e.g. organic solvents from chemical synthesis,^23^ all references samples were prepared and annealed in different purified-Ar glovebox systems. After preparation of the samples in the foreign purified-Ar glovebox system, they were mounted in a high-vacuum tight steel container (as backfilled with purified-Ar) and subsequently transferred to the HAXPES glovebox. The samples were typically transferred within about 20 min from the synthesis glovebox to the purified-Ar glovebox of the XPS/HAXPES system; next, the steel container was opened (under Ar shielding gas) and mounted on the sample platen for XPS/HAXPES analysis. As such, all possible measures were taken to prevent air-exposure and possible cross-contamination (e.g., from organic solvents^23^). The linearity of the energy scale of the hemispherical analyzer (i.e., from 0 eV up to 5400 eV) was calibrated according to ISO 15472 (Second edition 2010–05–01) by referencing the Au 4f^7^/_2_, Ag 3d^5^/_2_ and Cu 2p^3^/_2_ main peaks to the recommended binding energy (BE) positions of 83.96, 368.21, and 932.62 eV, respectively (as measured in situ for the sputter-cleaned, high-purity metal references using both the soft and hard X-ray source).^40^ The linearity of the energy scale over an extended energy range of 5400 eV varies between +0.1 eV and −0.3 eV, which corresponds to an accuracy of the energy scale linearity <0.01%: see Figure S5. The absolute error in the absolute BE values for XPS is about ±0.1 eV for a pass energy of 69 eV (ISO 15472 indicating ±0.07 eV for a pass energy of 50 eV),^40^ whereas the absolute error in the BE positions for HAXPES is about ± 0.17 eV (as estimated from the difference in peak positions of the Au 4f^7^/_2_ and Ag 3d^5^/_2_ peaks as measured by XPS and HAXPES for the pure Ag and Au metals): see Figure S6.

Charge neutralization during each measurement cycle was accomplished by dual-beam charge neutralization, employing low-energy electron and Ar ion beams (1-V bias, 20-μA current). All samples were fixed to a stainless-steel holder by sticky carbon tapes. First, XPS and HAXPES survey spectra, covering BE range of 0–1200 eV (XPS) and 0–5200 eV (HAXPES), were recorded with a step size of 0.5 eV at constant pass energy of 280 eV using the Al–Kα (power 51 W; beam diameter ~200 μm) and the Cr–Kα (power 100 W; beam diameter ∼150 μm) sources, respectively: see Figures S3 and S4, respectively. These settings correspond to a constant analysis area in the range of 200–280 μm^2^. Next, high-resolution spectra of the Li 1s, La 3d^5^/_2_, Zr 3d, C 1s and O 1s regions were measured with a step size of 0.05 eV at constant pass energy of 69 eV using both Al–Kα and the Cr–Kα sources. Notably, a pass energy of 69 eV was carefully selected, since it resulted in a similar energy resolution for both X-ray sources, as evidenced by a constant full-width-at-half-maximum of the Ag 3d^5^/_2_ peak of 0.9 eV for the XPS and HAXPES analysis. To enhance the signal from the interior of the oxide films and the bulk references, all XPS/HAXPES measurements were performed at a takeoff angle of 90° between the sample surface and the center of the entry lens of the analyzer (see Tables S1 and S2 in the Supporting Information).

Peak fitting was performed using the *CasaXPS* software, while applying a linear background for the O 1s, C 1s and Li 1s regions and a Shirley background for the La 3d^5^/_2_ and Zr 3d regions. The Gaussian fraction of each peak component was constrained to 0.5 (since tiny variations of this parameter had only a minor effect on the goodness of fit). The following principles were used for fitting of each peak: (*i*) the aliphatic C–C (adventitious) C 1s main peak was fixed at 284.8 eV to correct for charging, (*ii*) peaks associated with a specific chemical species were constrained at the same binding energy and full width at half-maximum for the entire data set (for instance, the C 1s peak position of Li_2_CO_3_ should have a constant value of BE of 290.08 eV even for different LLZO samples) and (*iii*) improving the fit by introducing additional peaks related to “physically unknown” spectral species was avoided (i.e., the absolute minimum of peak components was introduced for describing the entire data set). Due to the low signal intensity of the HAXPES C 1s peaks of the Li-ArSp sample, its charging correction was conducted by refereeing the O 1s peak of O–C=O to 532.7 eV.

The probing depths of the measured photoelectron lines correspond to 3λ×sin­(θ), where λ denotes the inelastic mean free path of the emitted photoelectrons traversing through the studied compound and θ is the detection angle with respect to the sample surface: see Tables S1 and S2 in the Supporting Information. Values of λ were calculated from the so-called TTP2 formalism^59^ using the QUASES-IMFP-TPP2M software (version 3; freely available at http://www.quases.com), while adopting the corresponding values for the density, bandgap and the number of valence electrons, as reported in Table S3.

**Powder XRD Measurements** were conducted using a STOE STADIP powder X-ray diffractometer in transmission mode using Cu–Kα irradiation with a wavelength of 1.5406 Å.

## Supplementary Material



## Notes

Additional experimental details, supporting Figures S1–7 and Tables S1–5. (PDF)
