# Supplementary material for: Standardizing XPS and HAXPES Analyses of LLZO Solid-State Electrolytes and Their Reactive Compounds
Source: ACS Mater Au. 2025 Jun 26;5(5):785–97. doi: 10.1021/acsmaterialsau.4c00174 (PMC12426779; doi:10.1021/acsmaterialsau.4c00174)
Supplement: Supplementary file 1 [file mg4c00174_si_001.pdf]

Supporting Information for

Standardizing XPS and HAXPES analyses of LLZO solid-state electrolytes and their reactive compounds

Huanyu Zhang,[1,2†] *Lars P.H. Jeurgens,*[3†*] *Claudia Cancellieri,*[3] *Jaka Sivavec,* [1,2] *Maksym V. Kovalenko,*[1,2*] *Kostiantyn V. Kravchyk,*[1,2*]

[1]Laboratory of Inorganic Chemistry, Department of Chemistry and Applied Biosciences, ETH Zürich, CH-8093 Zürich, Switzerland

[2]Laboratory for Thin Films and Photovoltaics, Empa - Swiss Federal Laboratories for Materials Science & Technology, CH-8600 Dübendorf, Switzerland

[3]Laboratory for Joining Technologies & Corrosion, Empa - Swiss Federal Laboratories for Materials Science & Technology, CH-8600 Dübendorf, Switzerland

† H.Z. and L.P.H.J. contributed equally to this work.

Corresponding Authors:

*E-mails: lars.jeurgens@empa.ch, Kostiantyn.Kravchyk@empa.ch

and mvkovalenko@ethz.ch

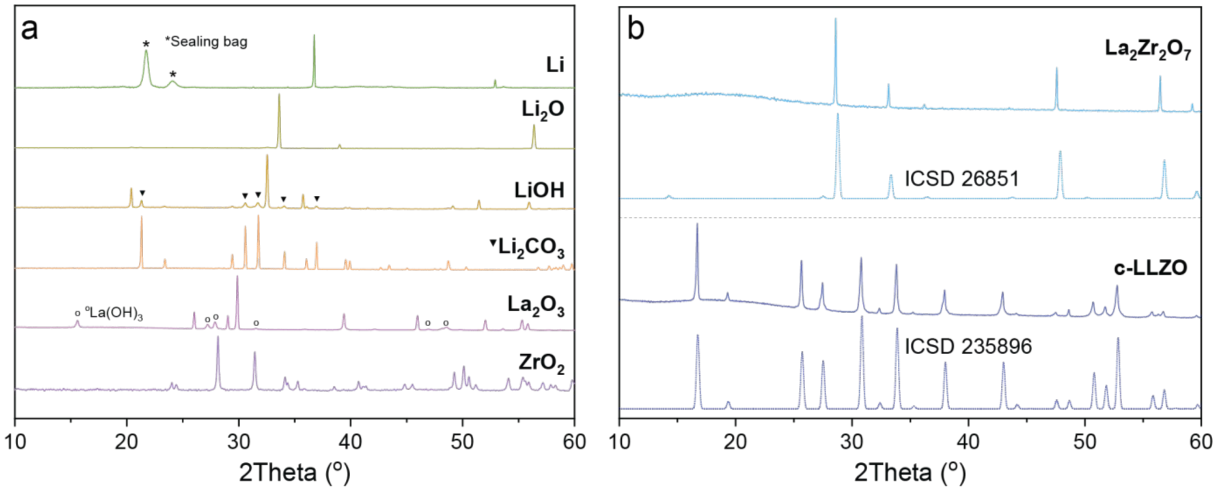

Figure S1. PXRD patterns of (**a**) the commercially purchased powders and (**b**) synthesized LZO and c-LLZO. The commercial powders were heat-treated at 100 °C overnight in vacuum oven installed in a purified-Ar glovebox. LiOH and La_2O_3 show minor impurities of Li_2CO_3 and $La(OH)_3$ respectively, due to reaction in air. Li metal was vacuum-sealed in plastic sealing bag for the XRD measurements, while other samples were filled into quartz capillaries. LZO and c-LLZO are synthesized and sintered respectively (see Experimental section). The corresponding diffractograms from the ICSD database are showing for comparison.

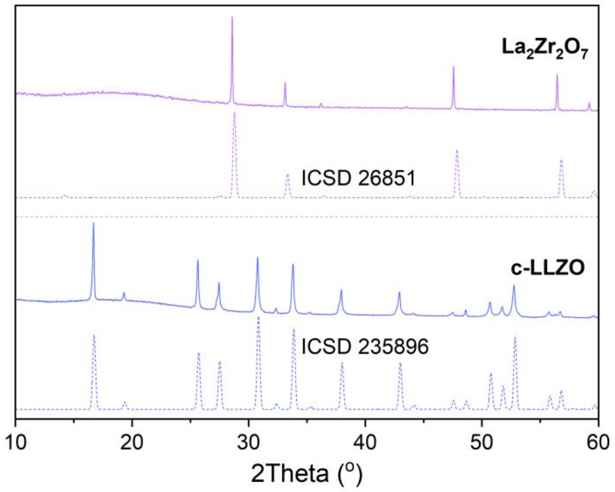

Figure S2. PXRD patterns of as-synthesized $La_2Zr_2O_7$ and as-sintered c-LLZO.

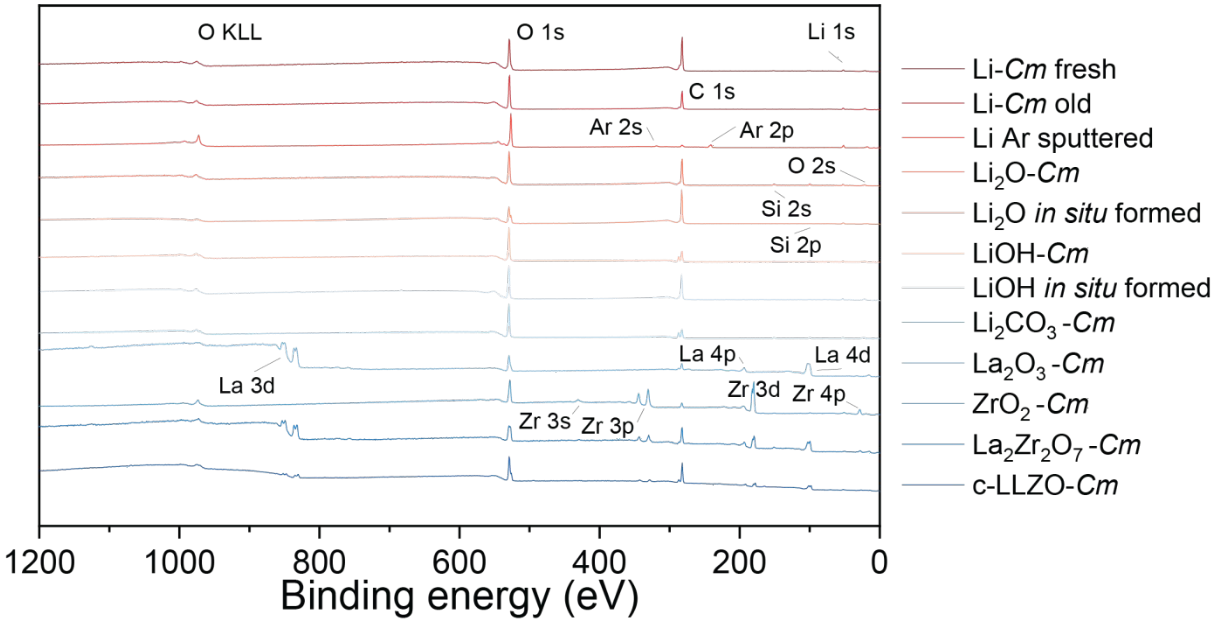

Figure S3. Measured XPS survey spectra of fresh Li, stored Li, Ar-sputtered Li, Li_2O, *in situ* formed Li_2O, LiOH, *in situ* formed LiOH, Li_2CO_3, La_2O_3, ZrO_2, $La_2Zr_2O_7$ and c-LLZO.

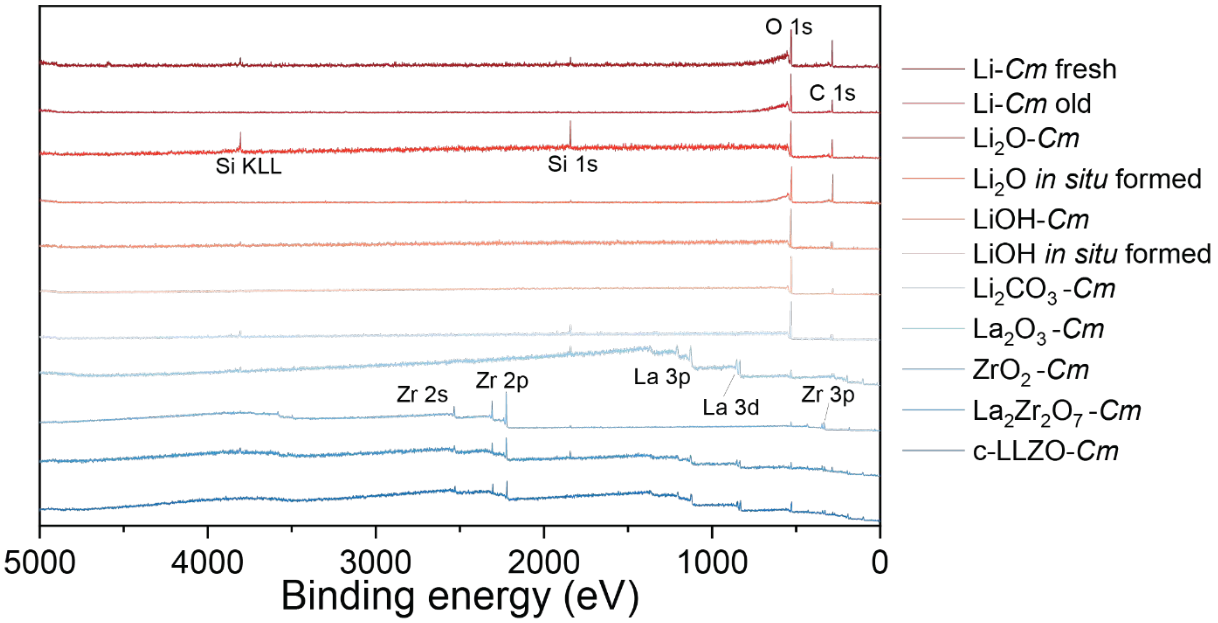

Figure S4. Measured HAXPES survey spectra of fresh Li, stored Li, Li_2O, *in situ* formed Li_2O, LiOH, *in situ* formed LiOH, Li_2CO_3, La_2O_3, ZrO_2, $La_2Zr_2O_7$ and c-LLZO.

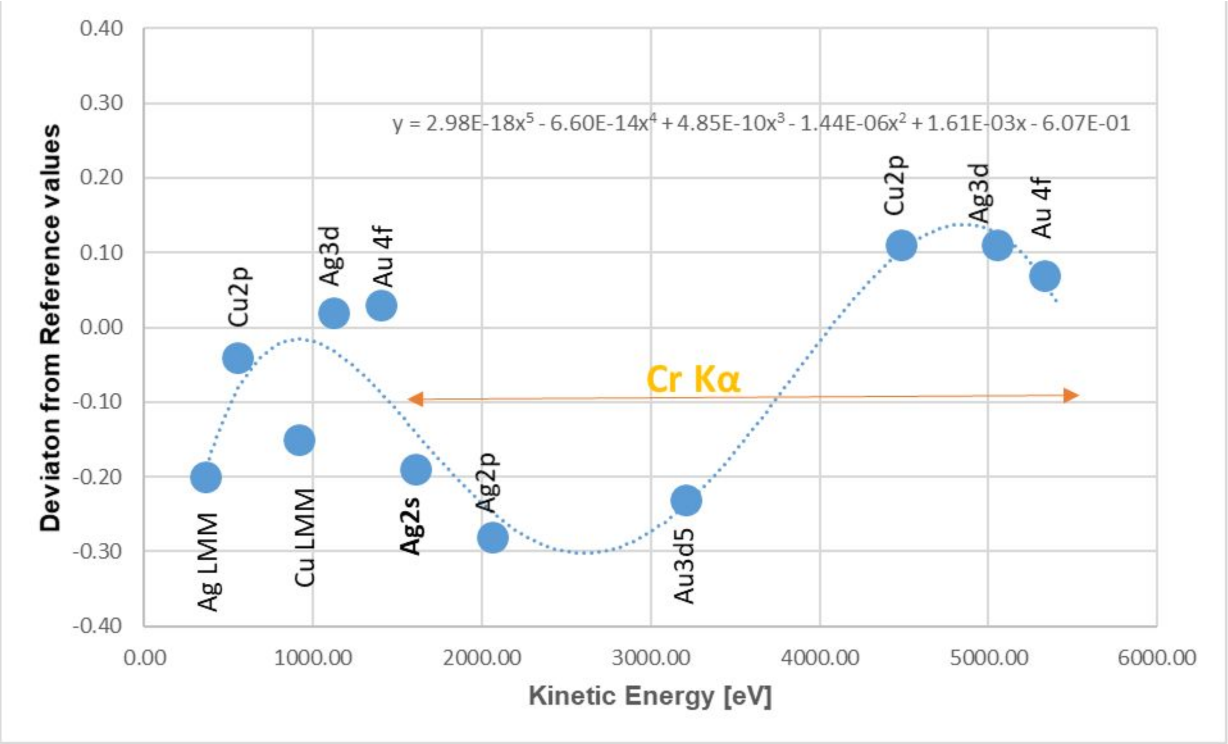

Figure S5. Deviation between recommended and measured kinetic energies values for various reference lines according to Ref. [1], as resulting after systematic correction of the work function and retard ratio of our lab-based XPS/HAXPES Quantes instrument. The maximum variation of the linearity of the energy scale over an extended energy range of 5400 eV is between +0.1 eV and -0.3 eV, which indicates that the energy scale linearity has an accuracy within < 0.01%. A polynomial fit allows precise corrections at the specific kinetic energy positions, if needed. Regular verification of the energy scale calibration indicates that the energy scale linearity deviation is very stable over time, while the absolute scale (i.e. work function) may slightly vary (especially after a bake-out).

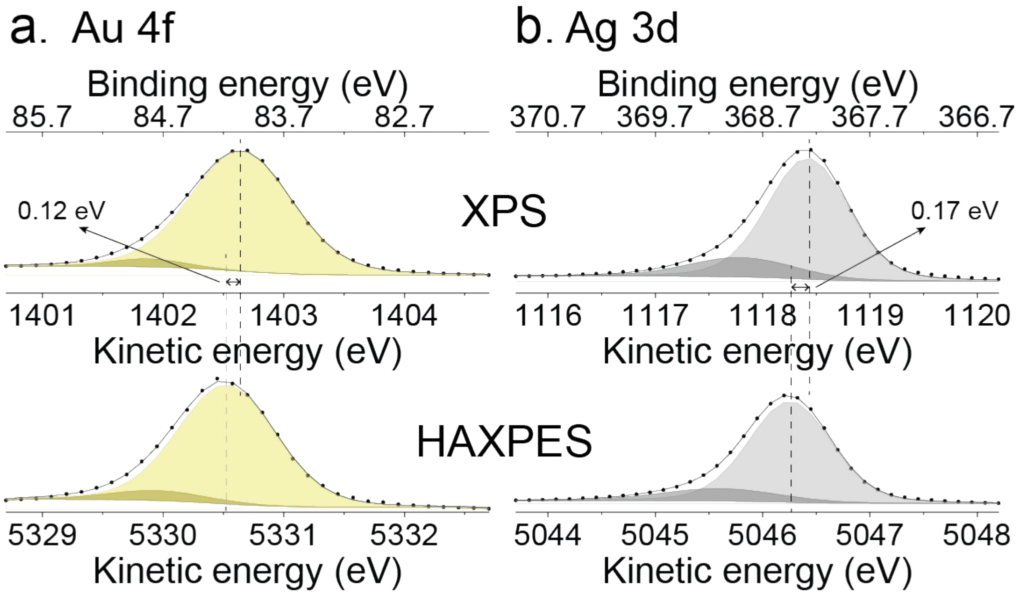

Figure S6. Au 4f$^7/_2$ (**a**) and Ag 3d$^5/_2$ (**b**) spectra of gold and silver reference samples as measured using Al-α (XPS) and Cr-α (HAXPES) radiation. This indicates a maximum error in the absolute BE values for the XPS and HAXPES reference spectra of Ag and Au metal of ± 0.17 eV.

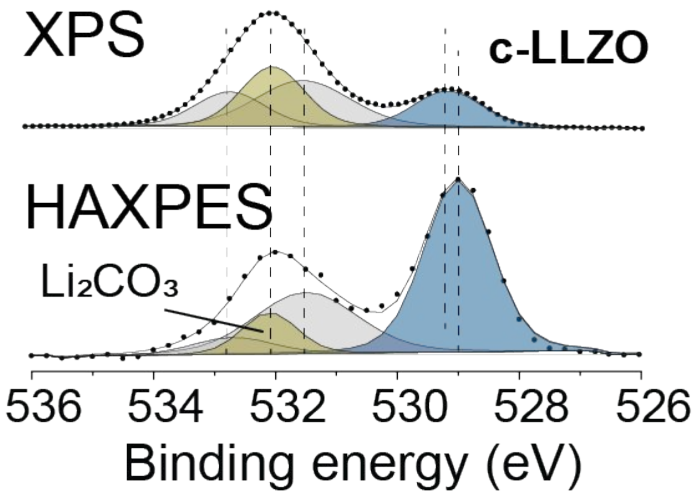

Figure S7. Reconstruction of the XPS and HAXPES O 1s spectra of c-LLZO (after charge correction) by introducing only a single instead of two main peaks in the BE range from 528 – 530 eV. It evidences a distinct shift of the fitted single main peak in the range from 528 – 530 eV towards lower BE values for a higher probing depth, as achieved by HAXPES. This justifies the introduction of two O 1s main peaks for c-LLZO in the fitted BE range of 528 – 530 eV, as assigned to O in the c-LLZO lattice and O in Li_2O, respectively.

Table S1. Probing depths of La 3d, O 1s, C 1s, Zr 3d and Li 1s photoelectrons emitted by Al-α radiation ($h\nu = 1486.7$ eV) and traversing through the indicated reference material, as detected at an angle θ with respect to the sample surface for XPS spectra. The probing depths correspond to $3\lambda \times \sin(\theta)$, where λ denotes the inelastic mean free path of the emitted photoelectrons traversing through the respective compound. Values of λ were calculated from the so-called TTP2 formalism[2] using the QUASES-IMFP-TPP2M software (version 3; freely available at http://www.quases.com), while adopting the corresponding values for the density, bandgap and the number of valence electrons, as reported in Table S3.

	**Probing depth (nm), 3λ×sin(θ), for XPS**					
**Compound**	**θ (°)**	**La 3d**	**O 1s**	**C 1s**	**Zr 3d**	**Li 1s**
**Li**	90		9.87	11.89		13.76
**Li_2O**	90		7.68	9.17		10.55
**LiOH**	90		8.13	9.72		11.20
**Li_2CO_3**	90		7.90	9.41		10.82
**La_2O_3**	90	4.34	5.75	6.84		
**ZrO_2**	90		5.52	6.56	6.99	
**$La_2Zr_2O_7$**	90	4.17	5.53	6.58	7.01	
**c-LLZO**	45	3.22	4.28	5.09	5.43	5.85

Table S2. Probing depths of La 3d, O 1s, C 1s, Zr 3d and Li 1s photoelectrons emitted by Cr-α radiation ($h\nu = 5414.7$ eV) and traversing through the indicated reference material, as detected at an angle θ with respect to the sample surface. See caption text of Table S1 for further details.

	**Probing depth (nm), 3λ×sin(θ), for HAXPES**					
**Compound**	**θ (°)**	**La 3d**	**O 1s**	**C 1s**	**Zr 3d**	**Li 1s**
**Li**	90		38.8	40.5		42.0
**Li_2O**	90		28.9	30.2		31.3
**LiOH**	90		30.9	32.2		34.1
**Li_2CO_3**	90		29.5	30.7		31.9
**La_2O_3**	90	20.0	21.1	21.9		
**ZrO_2**	90		20.2	21.1	21.4	
**$La_2Zr_2O_7$**	90	19.2	20.3	21.1	22.6	
**c-LLZO**	45	14.9	15.8	16.4	16.7	17.0

Table S3. Physical input data (i.e. density, number of valence electrons (Nv), atomic mass, and band gap) as required for calculating the IMFP to estimate the probing depths in Tables 1 and 2.

Compound	Density	Nv	Atomic mass	Band gap
	g cm^{-3}	-	-	eV
**Li**	0.5334	1	6.94	0
**Li_2O**	2.013	8	29.88	4.9
**LiOH**	1.46	8	23.95	4.0
**Li_2CO_3**	2.43	24	73.89	3.7
**La_2O_3**	6.51	24	325.81	3.8
**ZrO_2**	5.68	16	123.218	3.5
**$La_2Zr_2O_7$**	6.02	56	572.255	3.7
**c-LLZO**	5.1	96	841.279	6

Table S4 Full width at half maximum (FWHMs) of the fitted main peaks in the reconstructed La $3d^{5}/_{2}$, O 1s, C 1s, Zr $3d^{3}/_{2}$:$3d^{5}/_{2}$, and Li 1s XPS and HAXPES spectra pertaining, corresponding to Table 2. The FWHMs of the peaks from adventitious carbon species were allowed to vary between lower and upper bounds of 1 to 2 eV, respectively, while constraining their relative chemical shifts.

	Binding energies (eV)								
**Chemical State**	**X-ray Source**	**La $3d^{5}/_{2}$**			**O 1s**	**C 1s**	**Zr 3d**		**Li 1s**
		**f^0**	**f^1L_{ab}**	**f^1L_b**			**$3d^{5}/_{2}$**	**$3d^{3}/_{2}$**	
**Li**	Al-Kα								2.15
	Cr-Kα								2.65
**Li Ar sputtered**	Al-Kα				1.47				1.75
	Cr-Kα				1.46				2.30
**Li_2O**	Al-Kα				1.05				1.90
	Cr-Kα				1.42				2.15
**Li_2O *in situ***	Al-Kα				1.39				1.81
	Cr-Kα				1.43				2.47
**LiOH**	Al-Kα				1.34				1.63
	Cr-Kα				1.36				1.42
**LiOH *in situ***	Al-Kα				1.64				1.91
	Cr-Kα				1.42				2.16
**Li_2CO_3**	Al-Kα				1.50	1.56			1.69
	Cr-Kα				1.49	1.49			1.83
**La_2O_3**	Al-Kα	3.14	2.76	2.35	1.22				
	Cr-Kα	2.98	2.11	2.18	1.33				
**ZrO_2**	Al-Kα				1.49		1.49	1.49	
	Cr-Kα				1.41		1.34	1.34	
**$La_2Zr_2O_7$**	Al-Kα	2.16	2.18	2.18	1.37		1.54	1.54	
	Cr-Kα	1.86	2.19	2.04	1.33		1.47	1.47	
**c-LLZO**	Al-Kα	2.00	2.06	2.00	1.34		1.16	1.16	1.84
	Cr-Kα	2.00	2.00	2.00	1.73		1.21	1.21	2.03
**C-C (adv. C)**	Al-Kα					1-2			
	Cr-Kα								
**C-O (adv. C)**	Al-Kα				1-2	1-2			
	Cr-Kα								
**O-C=O (adv. C)**	Al-Kα				1-2	1-2			
	Cr-Kα								

Table S5 Energy splitting (difference) between the "reference" main peak component *i*, as resolved from the reconstructed La 3d$^5/_2$, C 1s, Zr 3d$^3/_2$:3d$^5/_2$, and Li 1s spectra, and the respective "reference" O 1s main peak component, as resolved from the corresponding reconstructed O 1s spectra. (BE_i - BE_{O1s})

		BE_i - BE_{O1s} (eV)						
**Chemical State**	**X-ray Source**	**La 3d$^5/_2$**			**C 1s**	**Zr 3d**		**Li 1s**
		**f^0**	**f^{1}L$_{ab}$**	**f^{1}L$_b$**		**3d$^5/_2$**	**3d$^3/_2$**	
**Li_2O**	Al-Kα							-473.72
	Cr-Kα							-473.87
**Li_2O *in situ***	Al-Kα							-474.80
	Cr-Kα							-474.80
**LiOH**	Al-Kα							-476.73
	Cr-Kα							-476.43
**LiOH *in situ***	Al-Kα							-476.27
	Cr-Kα							-475.94
**Li_2CO_3**	Al-Kα				-242.01			-476.67
	Cr-Kα				-241.78			-476.42
**La_2O_3**	Al-Kα	305.75	307.75	309.67				
	Cr-Kα	305.09	306.99	309.21				
**ZrO_2**	Al-Kα					-347.80	-345.44	
	Cr-Kα					-348.01	-345.64	
**$La_2Zr_2O_7$**	Al-Kα	304.66	306.71	309.19		-347.83	-345.49	
	Cr-Kα	304.35	306.45	309.01		-348.07	-345.71	
**c-LLZO**	Al-Kα	304.08	306.46	308.64		-348.56	-346.18	-474.12
	Cr-Kα	303.88	306.33	308.47		-348.82	-346.42	-474.31

REFERENCES

(1) Siol, S.; Mann, J.; Newman, J.; Miyayama, T.; Watanabe, K.; Schmutz, P.; Cancellieri, C.; Jeurgens, L. P. H. Concepts for chemical state analysis at constant probing depth by lab-based XPS/HAXPES combining soft and hard X-ray sources. *Surf. Interface Anal.* **2020**, *52* (12), 802-810.

(2) Tanuma, S.; Powell, C. J.; Penn, D. R. Calculations of electron inelastic mean free paths. V. Data for 14 organic compounds over the 50–2000 eV range. *Surf. Interface Anal.* **1994**, *21* (3), 165-176.
